# Assessment of Safety and Efficacy of Expanded Hemodialysis with Medium Cut-Off Dialyzer Compared to Haemodiafiltration

**DOI:** 10.3390/jcm14061798

**Published:** 2025-03-07

**Authors:** Matteo Marcello, Marco Simonini, Anna Lorenzin, Valentina Corradi, Grazia Maria Virzì, Carlotta Caprara, Alessandra Brendolan, Claudia Benedetti, Paolo Lentini, Monica Zanella, Claudio Ronco

**Affiliations:** 1 Department of Nephrology, Dialysis and Transplant, San Bortolo Hospital, 36100 Vicenza, Italy; 2 International Renal Research Institute of Vicenza, 36100 Vicenza, Italy; 3 Department of Nephrology, San Raffaele Hospital, 20132 Milan, Italy; 4 Department of Nephrology and Dialysis, San Bassiano Hospital, 36061 Bassano, Italy

**Keywords:** expanded haemodialysis, haemodiafiltration, medium cut-off, middle molecules

## Abstract

**Background:** Removal of large uraemic toxins is still a challenge. Haemodiafiltration (HDF) has produced some results, although large convective volume, optimal vascular access to increase the blood flow rate and strict water quality management are required. Medium cut-off, high-retention-onset membranes have been recently developed, introducing the concept therapy called expanded haemodialysis (HDx). Furthermore, vitamin E-coated membrane has potential beneficial effects on inflammation and oxidative stress. **Methods:** A prospective longitudinal multicentre study was conducted for 3 months among 24 chronic haemodialysis patients. Patients were randomly assigned into either HDF with high-flux membrane or HDx with Theranova or ViE-X membrane. The primary goal was to assess albumin loss among the three types of dialyzers. Secondary goals included assessment of depurative efficacy for uraemic toxins and clinical outcomes. **Results:** Mean albumin loss was significantly higher in patients undergoing HDx with Theranova membrane, without any difference in serum albumin concentration among the three groups. Instantaneous clearance of small and middle molecules was significantly higher in patients undergoing HDF, but we did not find differences in removal ratio and Kt/V. Reduction in the erythropoietin resistance index was observed in patients treated with ViE-X membrane due to their lower dialysis vintage. **Conclusions:** The higher albumin loss during HDx has no effects on pre-dialysis serum albumin. HDx with Theranova in the presence of lower session length, lower Qb, lower convective dose, and lower instantaneous clearance reached the same dialysis efficacy compared to HDF.

## 1. Introduction

Along with the retention of metabolic waste products, patients with advanced kidney disease typically experience a constellation of symptoms, which lead to a reduced quality of life and increased morbidity and mortality. Complications of uraemia in patients with adequate urea kinetics and Kt/V have been correlated with uraemic toxins in the molecular range of 5000–50,000 Da, which are not adequately cleared by current dialysis techniques [1]. These molecules have been shown to have significant effects on the cardiovascular system, inflammation, and fibrosis [2,3]. Myoglobin (molecular weight: 17 kDa) is involved in organ damage, oxidative stress, and mitochondrial dysfunction [4]. Restless leg syndrome (RLS) is a neurological complication often seen in HD patients related to the accumulation of α1-microglobulin, a middle molecule (molecular weight: 33 kDa) [5]. Patients with CKD have high plasma concentration of inflammatory markers such as C-reactive protein (CRP) and reactive oxygen species (ROS) [6]. Furthermore, the increased expression of proinflammatory cytokines such as IL-1, IL-6, and TNF-α leads to vascular calcification, oxidative stress, and endothelial dysfunction [7]. Increased oxidative stress, which is prominent in HD, has also been proposed as a significant link to uraemic myopathy and fatigue in renal failure [8].

Small molecules (<500 Da) are efficiently removed during the diffusion process, while the removal of middle and large molecules (50–15,000 Da) is still a challenge [9]. HDF has been developed to efficiently remove middle molecules as well as small solutes. However, patients treated with HDF require a large convective volume (>23 L/session) [10], optimal vascular access to increase the blood flow rate, and strict water quality management [11]. Despite the advanced technology and results achieved by HDF, clinical outcomes in dialysis are still suboptimal.

Recently, a new class of membranes called medium cut-off (MCO) dialyzers have shown to provide a greater efficiency in the removal of larger solutes with molecular weight up to 45 kD, with only marginal albumin loss [12]. These membranes, also called medium cut-off high retention onset, have a tight pore size distribution that was designed to have a steep sieving curve in order to minimize the molecular weight interval between the MWRO and the MWCO. In MCO membranes, the combination of hydraulic permeability and geometric structure of the fibres enhances the process of internal filtration and backfiltration [13]. The term expanded haemodialysis has been proposed to define a treatment where diffusion and convection are combined inside a hollow-fibre dialyzer equipped with a medium cut-off high-retention-onset membrane. The combination of increased pore size and pore density with an advanced sieving profile and increased internal filtration allows an enhanced clearance of large middle molecules [14].

The aim of this study was to evaluate safety and efficacy of expanded haemodialysis with the new membrane Vie-X^®^, comparing its performance with the analogous medium cut-off membrane and haemodiafiltration with high-flux membrane. The primary goal was to assess albumin loss among the three types of dialyzers. Secondary goals included assessment of depurative efficacy for small and middle molecules and the dialyzer’s effect on anaemia.

## 2. Materials and Methods

### 2.1. Study Design

The following represent a prospective, observational, single-centre study involving 18 patients and conducted over a 3-month period in the Dialysis Centre at San Bortolo Hospital in Vicenza, Italy.

Patients were assigned into three groups (six patients each):

- Group 1, HDx with Asahi Kasei Medical ViE-X
- Group 2, HDx Baxter Theranova 400;
- Group 3, HDF with Fresenius CorDiax Fx80.

ViE-X^®^ is a polysulfone, vitamin E-interactive membrane with a presumed pore diameter of 48 Ä, hollow fibre with a length of about 300 mm, and internal diameter of 185 μm, allowing advanced sieving profiles and increased internal filtration.

Dialysis prescription was not changed for Group 2 and Group 3, while Vie-X^®^ was assigned to patients in Group 1 previously treated with high-flux HD.

All patients in the study provided informed consent, and the study was approved on 8 June 2021 by the local Ethics Committee (*n*° 25/21) and was conducted according to the Declaration of Helsinki.

### 2.2. Patients’ Selection

The study included all patients aged 18 years, with stage 5 CKD in haemodialysis, and who provided informed consent. Exclusion criteria were liver disease, oncologic disease and lympho- or myeloproliferative disorders under chemotherapy, autoimmune disorders or immunosuppression state, pregnancy, and denial to give written consent for participation. Dialysis vintage was not considered in the selection of patients because we assessed only the short-term effect of the dialyzer.

Dialysis buffer with bicarbonate and dialysate flow 500 mL/min were present for all patients. The anticoagulation used was unfractionated heparin. Net fluid removal was set individually, depending on the patient’s clinical needs. Different dialysis monitors were used: Bellco Flexya, Baxter Artis Physio, Nikkiso DBB, and Fresenius 5008 and 6008.

### 2.3. Laboratory Sampling

We analysed four early-week HD sessions at the beginning of each month over a 3-month period:

- T0. First HD session analysed and first use of ViE-X dialyzer in Group 1;
- T1. After one month from the first HD session;
- T2. After two months from the first HD session;
- T3. After three months from the first HD session.

Throughout the treatment, a proportional part of the dialysis fluid was collected to quantify albumin loss.

Dialysis adequacy was assessed in terms of single-pool Kt/V and removal ratio (RR) of urea, creatinine, and phosphate. We evaluated the removal of small and middle molecules utilizing the removal ratio and instantaneous clearance.

Laboratory measurements carried out at the beginning and at the end of the dialysis session included blood count, urea, creatinine, phosphorus, uric acid, phosphorus, myoglobin, α1-microglobulin, β2-microglobulin, and IL-6. Furthermore, blood samples were taken from arterial and venous lines of the extracorporeal circuit in order to assay the instantaneous clearance (K) of small and middle molecules. Laboratory measurements for instantaneous clearance were divided into two intradialytic timepoints:

- Initial part of HD session (I), taken 15 min after the beginning of HD session;
- Final part of HD session (F), taken 15 min from the end of HD session.

The gap between the initial and final clearance of solutes was indicated as ΔK.

All laboratory measurements on blood and effluent fluid were performed at the Laboratory Medicine Department of San Bortolo Hospital in Vicenza, except for the dosage of α1-microglobulin and β2-microglobulin, which was performed at the Clinical Chemistry Department of University Hospital of Padova. Laboratory dosage of IL-6 was analysed at Research Laboratory in Nephrology Department of San Bortolo Hospital, Vicenza.

### 2.4. Calculations

Albumin loss throughout the treatment was evaluated from the albumin concentration in the dialysate and the total volume of effluent fluid.

For mixed diffusive–convective treatment as haemodiafiltration or bicarbonate dialysis in the presence of ultrafiltration, instantaneous clearance of a determined solute was calculated as follows:

K = [(Qpi × Cpi) − (Qpo × Cpo)]/Cpi

where Qpi is the effective plasmatic flow rate entering the dialyzer; Qpo is the plasmatic flow rate leaving the dialyzer and calculated as the difference between Qpi and Qf (Qpo = Qpi − Qf). Qf is the total ultrafiltration rate equivalent to the net ultrafiltration volume during bicarbonate dialysis treatment and calculated as the sum of the net ultrafiltration volume and the total substitution fluid volume during HDF treatment. Cpi and Cpo are the plasma water concentrations in the arterial and venous line, respectively.

For the assessment of urea instantaneous clearance blood flow, Qb was used. For all other molecules, plasma water flow rate (Qp) was used, calculated as follows: Qp = (1 − Hct) × Qb.

The Δ clearance was calculated as the difference between final and initial clearance values.

Removal ratio (RR) was calculated as follows:

RR = (C_pre_ − C_post_)/C_pre_

where C_pre_ and C_post_ are the plasma concentration of solutes measured prior and after the dialysis session, respectively.

Dialyzers’ effects on anaemia and ESA dosage were evaluated with the erythropoietin resistance index (ERI). ERI is defined as average weekly erythropoietin (EPO) dose per kg body weight (wt) per average haemoglobin (Hgb) over a 3-month period (ERI = (EPO/wt)/Hgb).

Basal ERI was calculated in the three-month period preceding the study to evaluate the effect of introduction of the ViE-X dialyzer on anaemia. The gap between ERI and basal ERI was called ΔERI.

### 2.5. Statistical Analysis

All statistical analysis was carried out using SPSS, version 23 (IBM Co., Armonk, NY, USA). Sample size was calculated as 18 patients, accepting a power of 80% and standard error α of 0.05, with effect size expressed as Cohen f = 0.75, which allowed to assess the primary endpoint. The Kolmogorov–Smirnov test was used to analyse the variables’ distribution patterns. The baseline clinical characteristics are expressed as the means ± standard deviations, numbers (percentage, %), and median/IQR depending on the nature of the variables. Differences in biochemical parameters and albumin loss among different timepoints were analysed using the repeated-measures one-way analysis of variance. Differences in albumin loss, small and middle molecules’ clearances, and removal ratio among different dialyzers were analysed using ANOVA test. Correlation between clinical, anthropometric, and biological parameters was carried out using bivariate correlation test.

## 3. Results

This prospective observational study was carried out in 18 patients (11 men and 7 women) with a mean age of 67.7 ± 7 years (range 52 to 79 years) who were stable on a thrice-weekly haemodialysis (average dialysis vintage of 82 months, interval 1–437 months). Dialysis vintage was higher in patients undergoing HDF (*p* = 0.042). Underlying renal diseases were undiagnosed nephropathy in ten patients, chronic glomerulonephritis in five patients, obstructive nephropathy in two patients, and polycystic kidney disease in one patient. All participants were either anuric or oliguric (<500 mL/24 h), except for three patients (two patients in Group 1 and one in Group 2). Comorbidity was studied with Charlson index and incidence of diabetes. Thirteen patients had arteriovenous fistula, and five had a tunnelled central venous catheter.

Baseline characteristics are summarised in Table 1.

We analysed four early-week HD sessions at the beginning of each month over a 3-month period. Dialysis time was 210 min. Convective volume (Qf) during bicarbonate haemodialysis was calculated as the net ultrafiltration volume. During HDF treatment, convective volume was calculated as the sum of net ultrafiltration volume and the total replacement volume. As shown in Table 1, Qb and Qf values differ between the three filters, and it is higher in patients undergoing HDF (*p* < 0.001).

### 3.1. Primary Endpoint

In all four HD sessions analysed, albumin loss was different among the three dialyzers (ANOVA *p* <0.001), even after correction for covariates (univariate analysis: Qb, Qf, BMI, and pre-dialysis serum albumin). Patients in Group 2 had significantly higher albumin loss than those in Group 3 (ANOVA, post hoc test *p* < 0.001) and those in Group 1 (ANOVA, post hoc test *p* = 0.006). No significant differences were detected between Group 1 and 3.

The introduction of the Vie-X dialyzer in Group 1 lead to an initial fall followed by a progressive increase in pre-dialysis serum albumin. No significant differences in the serum albumin trend throughout different timepoints were found among the three groups (analysis of repeated measures).

**Figure 1:**
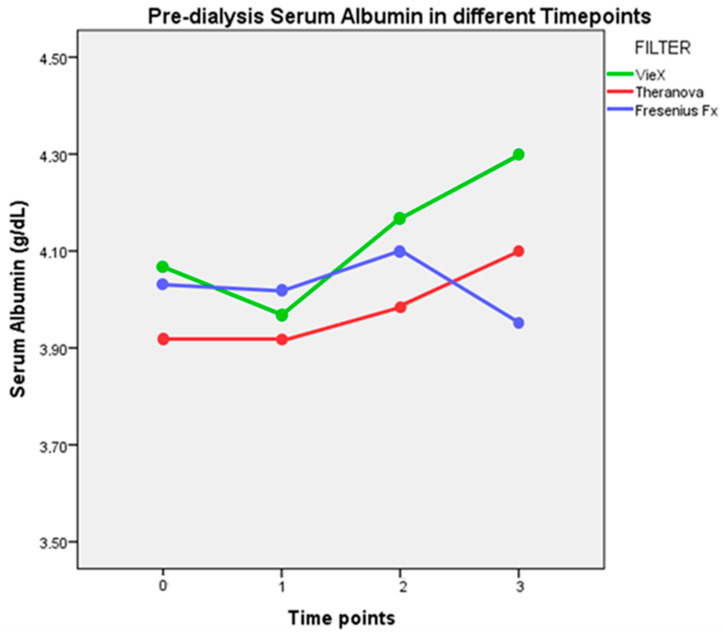
Serum albumin at different timepoints. Timepoint 0 refers to the first observation, and subsequent timepoints (1–3) refer to month from the first observation.

Mean albumin loss and pre-dialysis serum albumin values are summarized in Table 2 and Figure 1.

### 3.2. Secondary Endpoint

We found no differences in Kt/V between the three groups (Group 1, 1.3 ± 0.16; Group 2, 1.27 ± 0.17; Group 3, 1.29 ± 0.1). We found no significant differences in the removal ratios of small molecules and phosphate (Table 3).

The RR of α1-microglobulin in patients in Group 3 was significantly higher than in Group 1 (ANOVA *p* = 0.02) but not compared to Group 2. The RR of myoglobin was significantly higher in patients in Group 2 than in those in Group 1 (ANOVA, *p* = 0.04) and those in Group 3 (*p* = 0.05). Pre-dialysis α1-microglobulin serum concentration was significantly higher in Group 3 (ANOVA *p* < 0.001). Final α1-microglobulin serum concentration in patients in Group 3 was significantly higher than those in Group 2 (post hoc test *p* = 0.003). Patients in Group 2 had an initial serum myoglobin that was significantly higher (ANOVA, *p* = 0.005). No difference in final serum myoglobin was found among the groups.

Serum concentration before and after dialysis as well as removal ratios are summarized in Table 4.

Instantaneous clearance of urea, creatinine, and middle molecules was higher in Group 3. No difference was found in instantaneous clearance of phosphate between Group 2 and Group 3, but it was significantly lower in Group 1 compared to other groups. Instantaneous clearance values are summarized in Table 5.

We found no variation in haemoglobin among different groups and different timepoints (ANOVA). We found no differences between ERI and basal ERI and ΔERI values among the three groups. Furthermore, we found a reduction in ERI value in patients in Group 1, but it was associated with their lower dialysis vintage (bivariate correlation *p* = 0.009).

## 4. Discussion

In this prospective single-centre study, we made a cross-over comparison of 18 chronic haemodialysis patients undergoing either HDF or HDx. Patients were assigned into three group (six patients each): bicarbonate dialysis with either VIE-X (Asahi Kasei Medical) or Theranova 400 (Baxter) and haemodiafiltration HDF with Fresenius CorDiax Fx80.

The present clinical trial examined the short-term effects of the newly developed MCO dialyzer Vie-X in terms of safety and efficacy, comparing it with analogous MCO membrane Theranova and high-flux membrane Fresenius-Fx in HDF modality.

The primary aim of this study was to investigate the safety of different dialyzers in terms of albumin loss. In the evaluation of safety, we found in both dialysis modalities a mean albumin loss within 5 g per HD session, a cut-off above which a membrane is considered not suitable for chronic haemodialysis [15]. Mean albumin loss was significantly higher in patients undergoing HDx with Theranova membrane, while non-significant differences were observed among the other two membranes. During dialysis, a certain degree of albumin leakage is acceptable in order to remove larger molecules and protein-bound uraemic toxins without incurring the risk of low serum albumin levels. A recent prospective, randomized, crossover trial comparing high-flux haemodialysis with expanded haemodialysis showed a greater removal of large uraemic toxins using a medium cut-off dialyzer without excessive albumin leakage, which was in the mean range of 2.8 and 3.0 g per treatment [12]. In our study, the higher albumin loss observed in Group 2 did not influence serum albumin concentration. Similar to our report, a multicentre, randomized, controlled trial comparing the safety and efficacy of HDx with Theranova membrane and high-flux HD showed an albumin loss of 4 g per treatment while maintaining a constant serum albumin level over a period of 24 weeks [16]. Low serum albumin represents a strong predictor of mortality in haemodialysis patients but only when associated with chronic inflammation and decreased synthesis in malnourished patients [17], while the consequences of increased albumin loss during dialysis remains unknown, and its potential benefits due to the removal of protein-bound toxins are still under investigation [18]. In our study, as the dialysis prescription was not changed for Group 2 and Group 3, we did not expect to see any variation in serum albumin concentration during the 3-month observation period. In Group 1, with the introduction of MCO membrane, we observed an initial decrease in serum albumin concentration, followed by a gradual increase. However, no significant differences in the trend of serum albumin through different timepoints (analysis of repeated measures) were found between the three dialyzers. In previous clinical trials conducted for three months or less, pre-dialysis albumin levels were reduced after application of medium cut-off dialyzers [19]. However, different multicentre trials conducted over a period of 6 months or more showed that in patients previously treated with high-flux dialysis, the application of medium cut-off dialyzers led to an initial decrease in pre-dialysis albumin level, followed by a rise back to baseline [16,17,18,19,20]. This paradoxical behaviour usually observed at a timepoint between three to six months might be associated with the reduced inflammation and increased removal of uraemic toxins that occurs during expanded haemodialysis in comparison to high-flux haemodialysis.

The secondary endpoint of the study was dialysis efficacy in terms of removal of small and middle molecules. Instantaneous clearance of small solutes, including urea, creatinine, and uric acid, was significantly higher in patients undergoing haemodiafiltration. Similarly to previous study [12], we found no differences in the removal of small solutes as evaluated by the reduction ratio (RR). Furthermore, Kt/V values were above the minimum target of adequacy (Kt/V ≥ 1.2) [21] in all patients, without significant differences among the three groups.

The results from our analysis show that instantaneous clearance of both small and middle molecules is significantly higher in patients receiving haemodiafiltration.

Instantaneous clearance is strictly dependent on convective volume and thus on ultrafiltration rate (Qf). In patients undergoing expanded haemodialysis, the convective flux was equivalent to the net ultrafiltration rate set up according to interdialytic weight gain. In patients undergoing haemodiafiltration, the convective flux was calculated as the sum of net ultrafiltration rate plus the volume of substitution fluid; therefore, in our study, ultrafiltration rate (Qf) was significantly higher in patients in Group 3 receiving HDF. This largely explains the significant difference in instantaneous clearance values. However, during HDx, the initial and final instantaneous clearance values of β2-microglobulin and α1-microglobulin were similar, while during HDF, we observed a decrease in the instantaneous clearance throughout the dialysis session. Accordingly, we found the Δ clearance value of β2-microglobulin and α1-microglobulin significantly lower in patients undergoing HDF. This reduction in clearance value in Group 3 could be explained by the membrane fouling and concentration polarization occurring during HDF due to the higher ultrafiltration rate [22]; however, the same pattern was not observed for other uraemic toxins. A certain degree of convective flux independent from the net ultrafiltration rate usually takes place inside the MCO-HRO membrane. This phenomenon, known as internal-filtration/backfiltration, was demonstrated for the Theranova membrane by Lorenzin et al. [23], while it can be only hypothesized for Vie-X membrane. However, the main advantage of HDx is that the removal of middle molecules is dependent on the high sieving coefficient rather than the convective volume, and therefore, it can be used in patients with suboptimal vascular access and lower blood flow Qb. In our study, the majority of patients undergoing HDF (five out of six patients) had an arteriovenous fistula as a vascular access, and their Qb value was significantly higher compared to those receiving HDx, while half of patients undergoing HDx with Theranova membrane had a catheter as a vascular access. Blood flow Qb influences the performance of dialysis by increasing diffusive and convective dialysis dose and reducing the risk of coagulation with no additional costs [15]. A prospective single-centre study by Maduell et al. [24] comparing the safety and efficacy of HDx with high-flux HD and HDF at pre- and postdilution using a Qb of 250 mL/min showed that HDx achieved a better depurative performance than HD and pre-HDF but lower depuration than post HDF. In another study by Maduell et al. [25], increasing the Qb to 400 mL/min was associated with increased solute removal in both HDx and HDF, but the benefit was greater in HDF, probably because increasing the Qb facilitates a higher convective dose. In our study, even if instantaneous clearance values of middle molecules were higher in patients undergoing HDF, the removal ratio of myoglobin was significantly higher in patients receiving HDx with Theranova membrane. The same pattern was observed for β2-microglobulin, where the removal was higher with Theranova membrane, although no significant difference was found compared to HDF. Therefore, HDx with Theranova in presence of lower Qb and in the absence of a high convective dose reached a similar target of dialysis adequacy, with lower instantaneous clearance of middle molecules but without differences in the serum concentration of uraemic toxins and RRs. The long-term prognosis of HDx in comparison to HDF was evaluated by the CARTOON study [26], a recent multicentre randomized, controlled trial showing that HDx was not inferior to HDF in terms of cardiovascular risk.

Patients in Group 3, treated with HDF, had higher dialysis vintage compared to other groups. The only parameter assessed that could have been influenced by dialysis vintage was pre-dialysis serum concentration of uraemic toxins. However, we found significant differences only in the concentration of α1-microglobulin, which was higher in Group 3.

Expanded haemodialysis with ViE-X dialyzer was associated with lower pre-dialysis creatinine values, but this was probably related to the lower dialysis vintage of patients in Group 1. Mean albumin loss was 2.9 g in patients receiving HDx with ViE-X membrane, with no significant differences compared to patients undergoing HDF. Moreover, the introduction of ViE-X dialyzer in patients previously treated with high-flux HD was not associated with a decrease in pre-dialysis serum albumin. In the evaluation of the efficiency of ViE-X membrane, is necessary to take into account different features regarding patients in Group 1. The majority of patients in this group had an arteriovenous fistula (five out of six patients). Mean dialysis vintage was significantly lower compared to other groups, and only one patient receiving Vie-X membrane was anuric; however, blood flow (Qb) was significantly lower. Instantaneous clearance values were significantly lower compared to HDF but similar to those obtained with Theranova membrane. Even if instantaneous clearance of phosphate was significantly lower in patients receiving HDx with VIE-X membrane, the mean removal ratio of phosphate was similar to that achieved with Theranova. Serum concentration of α1-microglobulin increased during the dialysis session in patients receiving ViE-X membrane, probably due to a low permeability of the dialyzer for this large middle molecule (MW 33 KDa).

Different meta-analysis indicated that vitamin E-coated dialyzer can induce reductions in serum CRP, IL-6, and thiobarbituric acid reactive substance levels in patients receiving haemodialysis [27,28]. This effect of vitamin E-coated membrane seems to take place during the dialysis treatment but not during the interdialytic interval [29]. In our study, even if there was not a significant difference in the clearance value of IL-6 between Group 1 and Group 2, mean values were higher in patients undergoing HDx with ViE-X membrane. However, we found no significant difference in serum concentration or Δ IL-6 between the three groups. We also assessed the potential role of vitamin E-coated membrane in correcting anaemia using ERI. We found that the introduction of ViE-X membrane was associated with a progressive reduction in ERI from basal value in a 3-month-long therapy. Therefore, ΔERI was higher in comparison with other groups of patients, although it correlated significantly with dialysis vintage but was not influenced by the dialyzer.

One of the strengths of our study was the analysis of four dialysis sessions with each dialyzer. This allowed us to investigate the short-term performance as well as middle-term effects of three dialyzer and two different dialysis modalities.

A limitation of this study was the small sample size, which could have influenced some of our results. Furthermore, the dialysis vintage was significantly lower in the group of patients receiving the novel ViE-X membrane, and this could have influenced some biological biomarkers. Furthermore, in this study, a comparison with standard high-flux haemodialysis was not performed.

## 5. Conclusions

To our knowledge, this is one of the few studies that provides data on short- and middle-term outcome in patients receiving expanded haemodialysis compared to haemodiafiltration. Using three dialyzers with no significant differences in dialysis adequacy, we found that expanded haemodialysis with medium cut-off membranes without the use of a high volume of substitution fluids for the convective dose reached dialysis efficacy in terms of in terms of middle molecules removal and uraemic toxins serum concentration. Furthermore, the present study indicates that even if albumin loss is higher during HDx compared to haemodiafiltration, it has no effects on pre-dialysis serum albumin. However, these data need to be validated by more clinical trials with a larger sample size. Moreover, the long-term biological and clinical benefit of expanded haemodialysis remains to be investigated.

## Figures and Tables

**Table 1:** Descriptive analysis.

	HDx		HDF	*p*-Value
**Group**	**1**	**2**	**3**	
Filter	ViE-X	Theranova	Fresenius CorDiax	
Nr of patients (*n*/%)	6/25	6/25	6/25	
AGE (mean ± SD)	67.6 ± 4.1	65.17 ± 7.73	70.33 ± 10.1	ns
SEX (male) (*n*/%)	4/66.7	4/66.7	3/50	ns
BMI (mean ± SD)	27.3 ± 4.3	24.67 ± 5.68	22.82 ± 1.64	ns
URINE OUTPUT (*n*/%)				ns
<100 mL	1/16.7	1/16.7	5/83.3	
<500 mL	3/50	4/66.7	1/16.7	
>500 mL	2/33.3	1/16.7	0	
DIABETES (*n*/%)	2/33.3	1/16.7	3/50	ns
CHARLSON > 6 (*n*/%)	3/50	2/33.3	3/50	ns
CHARLSON (mean ± SD)	7.1 ± 0.9	5.67 ± 2.16	6.83 ± 2.99	ns
CKD CAUSE (*n*/%)				ns
UNKNOWN	4/66.7	1/16.7	5/83.3	
GLOMERULAR	2/33.3	3/50	0	
OBSTRUCTIVE	0	1/16.7	1/16.7	
ADPKD	0	1/16.7	0	
DIALISYS VINTAGE (months) (median/IQR)	15 [2.5–42.5]	91 [18.5–113.2]	149.8 [37–100]	0.042
VASCULAR ACCESS (*n*/%)				ns
AVF	5/83.3	3/50	5/83.3	
CVC	1/16.7	3/50	1/16.7	
Qb (mL/min) (median/IQR)	290 [281–300]	300 [287–300]	300 [300–350]	<0.001
Qf (mL/min) (median/IQR)	13.15 [12.3–14]	11.8 [7.9–14.3]	99.1 [85.9–108.2]	<0.001

**Table 2:** Effect of different treatment on Albumin loss.

Mean Albumin Loss (g)	
Vie-X	2.9
Theranova	3.7
Fresenius-Fx	2.5

**Table 3:** Removal ratios of small molecules. Kt/V has been calculated with ionic dialysance (ID).

	Vie-X	Theranova	Fresenius Fx	*p*-Value
RR Creatinine (Mean ± SD)	65.5 ± 6.3	66.3 ± 6.3	66.9 ± 5.1	ns
RR Phosphate (Mean ± SD)	58.2 ± 11.2	56.1 ± 9.4	60.2 ± 7.3	ns
RR Urea (Mean ± SD)	72.9 ± 5.1	72.1 ± 6.2	75.0 ± 4.4	ns
Kt/V (ID) ∗ (Mean ± SD)	1.30 ± 0.16	1.27 ± 0.17	1.29 ± 0.10	ns

**Table 4:** Removal of middle molecules. Pre is the pre-dialysis concentration of serum toxins. Post is the post-dialysis concentration of serum toxins.

		ViE-X	Theranova	Fresenius	*p*-Value
InterLeukin-6 (Mean ± SD)	Pre	6.93 ± 0.15	6.96 ± 0.17	6.88 ± 0.16	ns
	Post	6.86 ± 0.28	6.90 ± 0.17	6.88 ± 0.07	ns
	RR	0.9 ± 3.7	0.9 ± 2.6	−0.05 ± 2.9	ns
β2-microglobulin (Mean ± SD)	Pre	13.7 ± 2.8	16.0 ± 2.9	16.5 ± 3.6	ns
	Post	6.9 ± 1.1	7.2 ± 2.2	7.5 ± 1.8	ns
	RR	48.2 ± 8.9	55.9 ± 9.3	55.7 ± 13.2	ns
α1-microglobulin (Mean ± SD)	Pre	222.4 ± 54.1	212.7 ± 25.0	264.5 ± 22.0	<0.001
	Post	232.4 ± 57.9	211.7 ± 33.1	254.0 ± 46.8	0.012
	RR	−4.5 ± 5.8	0.7 ± 13.2	4.5 ± 12.9	0.024
Myoglobin (Mean ± SD)	Pre	196.4 ± 86.2	266.6 ± 121	198.2 ± 73.8	0.005
	Post	118.9 ± 46.5	135.2 ± 47.1	123.2 ± 73.5	ns
	RR	38.1 ± 8.6	47.1 ± 9	38.6 ± 23.1	0.07

**Table 5:** Instantaneous clearance of uraemic toxins.

Uraemic Toxins (Mean ± SD)	ViE-X	Theranova	Fresenius	*p*-Value
Urea	258 ± 16	258 ± 15	277 ± 29	0.003
Creatinine	133 ± 15	145 ± 13	151 ± 20	0.001
Interleukin-6	23.1 ± 17.4	12.8 ± 8	107.0 ± 28.1	<0.001
β2-microglobulin	75.4 ± 12.6	86.9 ±10.1	128.5 ± 32.4	<0.001
α1-microglobulin	8.8 ± 5.3	8.0 ± 7.3	101.6 ± 42.1	<0.001
Myoglobin	58.3 ± 9.8	69.7 ± 11.9	94.5 ± 46.1	<0.001

## Data Availability

Such dataset may be requested by email to Correspondent Author.
